# Simple scoring model for predicting overt hepatic encephalopathy in geriatric cirrhosis: A multicenter retrospective cohort study

**DOI:** 10.1007/s11011-025-01691-x

**Published:** 2025-09-10

**Authors:** Yuki Utakata, Takao Miwa, Masashi Aiba, Shinji Unome, Tatsunori Hanai, Kenji Imai, Yohei Shirakami, Koji Takai, Makoto Shiraki, Naoki Katsumura, Masahito Shimizu

**Affiliations:** 1 Department of Gastroenterology, Chuno Kosei Hospital, Seki, Gifu, 5-1 Wakakusa- dori, 501-3802 Japan; 2 Department of Gastroenterology/Internal Medicine, Graduate School of Medicine, Gifu University, Gifu, 1-1 Yanagido, 501-1194 Japan

**Keywords:** Albumin, Ammonia, Covert hepatic encephalopathy, Liver cirrhosis, Minimal hepatic encephalopathy

## Abstract

Identifying the risk of overt hepatic encephalopathy (OHE) in geriatric patients with cirrhosis remains challenging. This study aimed to investigate the independent factors for OHE development in geriatric cirrhosis and to establish a simple scoring model to identify individuals at risk for OHE. We conducted a retrospective review of geriatric patients with cirrhosis aged ≥ 80 years who were admitted between April 2006 and November 2022. Baseline parameters were assessed at the time of admission, and factors associated with OHE development were examined using Fine-Gray proportional hazards regression analysis, with mortality as a competing risk. Based on the factors associated with OHE development, a simple hepatic encephalopathy (sHE) score was calculated, and its efficacy was subsequently verified. Of the 270 patients analyzed, the median age was 83 years, and 63% were male. During a median follow-up of 1.8 years, 41 (15%) patients developed OHE and 120 (44%) patients died. Multivariable analysis revealed that serum albumin (sub-distribution hazard ratio [SHR], 0.51; 95% confidence interval [CI], 0.27–0.98; *p* = 0.042) and ammonia (SHR, 1.01; 95% CI, 1.00–1.02; *p* = 0.006) levels were independent factors for OHE development in geriatric cirrhosis. Based on the sHE score, the high-risk and intermediate-risk groups exhibited a high incidence of OHE, whereas those in the low-risk group rarely developed OHE. Serum albumin and ammonia levels were identified as independent risk factors for the development of OHE in geriatric cirrhosis. The sHE score was useful for stratifying the risk of OHE in the geriatric population.

**Supplementary Information:**

The online version contains supplementary material available at 10.1007/s11011-025-01691-x.

## Introduction

Hepatic encephalopathy (HE) is a significant complication of cirrhosis, with symptoms ranging from non-specific neurological or psychological abnormalities (covert HE [CHE]) to disorientation or asterixis (overt HE [OHE]) (Vilstrup et al. 2014; Bajaj et al. 2020; European Association for the Study of the Liver 2022). OHE is a common complication in patients with cirrhosis and has an annual incidence rate of approximately 10%, increasing with disease progression (Louissaint et al. 2022). Upon the occurrence of OHE in patients with cirrhosis, individuals experience poor quality of life, hospitalization, increased morbidity, and high mortality rates (Vilstrup et al. 2014; Bajaj et al. 2020; European Association for the Study of the Liver 2022). In addition, OHE not only affects the patients themselves but also has an impact on healthcare resource requirements, causing a socioeconomic burden and having a negative impact on their families and caregivers (Rose et al. 2020). Although several treatments for OHE have been proposed, patients often experience recurrence even during active therapy (Vilstrup et al. 2014). Therefore, there is an urgent need to identify patients at high-risk of developing OHE, monitor these patients closely, and control the underlying liver disease to prevent OHE and improve outcomes (European Association for the Study of the Liver 2022). 

Life expectancy has doubled over the last 200 years (Partridge et al. 2018). Subsequently, the average age of patients with cirrhosis in Japan has also increased, from 66.4 years before 2007 to 67.9 years between 2018 and 2021, which is consistent with the global trend (Enomoto et al. 2020, 2024; Louissaint et al. 2022). Age itself is a significant predisposing factor for liver fibrosis and is included as an important factor in the FIB-4 index, which is a widely used non-invasive marker for estimating liver fibrosis (Blanco-Grau et al. 2021). As aging is associated with neuropsychiatric functional decline conditions, such as dementia (Partridge et al. 2018), the diagnosis of CHE and risk stratification for OHE are challenging in the daily management of geriatric cirrhosis (Blanco-Grau et al. 2021). CHE tests and medical imaging techniques may assist in diagnosing and differentiating CHE to help identify patients at high-risk of OHE in geriatric cirrhosis (Kawaguchi et al. 2017; Tamai et al. 2022). However, these methods are limited by poor availability, time consumption, cost, and lack of specific cut-off values for the geriatric population. Therefore, there is a need to develop simple clinical assessment methods to help identify patients at high-risk of developing OHE, particularly in geriatric cirrhosis. 

We have previously developed a simple HE (sHE) score using serum albumin and ammonia levels to identify the risk of developing CHE and OHE in patients with cirrhosis (Miwa et al. 2022). However, few attempts have been made to develop a risk score to stratify the risk of OHE in geriatric cirrhosis. The primary objective of this study was to elucidate the independent factors for the development of OHE in geriatric cirrhosis, particularly in patients aged ≥ 80 years. The secondary objective was to establish a simple scoring model based on daily available variables to identify those at risk for OHE in geriatric cirrhosis.

## Methods

### Study design and participants

This multicenter retrospective cohort study included patients with geriatric cirrhosis aged ≥ 80 years who were admitted to the Chuno Kosei Hospital or the Gifu University Hospital between April 2006 and November 2022. Cirrhosis was diagnosed based on liver histology, laboratory data, and diagnostic imaging. Exclusion criteria were a history of OHE, uncontrolled hepatocellular carcinoma, other malignancies, heart failure, ongoing dialysis, respiratory failure, excessive alcohol consumption defined as > 60 g/day for males and > 50 g/day for females within a month (European Association for the Study of the Liver (EASL); European Association for the Study of Diabetes (EASD); European Association for the Study of Obesity (EASO). 2024), missing data, and refusal to opt-out. The study protocol was reviewed and approved by the Institutional Review Board of Gifu University Graduate School of Medicine (approval number: 2024–071). This study was conducted in accordance with the 2013 Declaration of Helsinki. Owing to the retrospective nature of the study, an opt-out method was used to obtain informed consent from the patients.

### Outcome and follow-up

The primary outcome of this study was the first episode of OHE in geriatric patients with cirrhosis. OHE was diagnosed by a hepatologist based on West Haven criteria during inpatient or after discharged in the outpatient clinic, defined as grade ≥Ⅱ based on clinical symptoms (Vilstrup et al. 2014). Patients diagnosed with OHE were hospitalized and received intravenous branched-chain amino acids followed by lactulose and/or rifaximin therapy. Mortality was also recorded, as it was considered a competing risk for OHE development. OHE and death were recorded until the patient’s last visit, death, or December 26, 2023, whichever occurred first. Patients were followed up in the outpatient clinic at least every three months after discharge and were treated according to the Japanese guidelines for cirrhosis (Yoshiji et al. 2021a, 2021b). Specifically, patients with active hepatitis C infection were treated with direct-acting antivirals and those with active hepatitis B infection were managed with nucleoside analogs in accordance with the guideline (Drafting Committee for Hepatitis Management Guidelines, the Japan Society of Hepatology 2020a, 2020b).

### Data collection and the simple HE score

The following baseline data were collected from the medical records: age, sex, weight, height, etiology of cirrhosis, comorbidities (including diabetes mellitus, ascites, previous OHE, and hepatocellular carcinoma), medications and laboratory data. Body mass index, Child-Pugh, and Model for End-Stage Liver Disease (MELD) scores were calculated from the obtained data. The bilirubin-albumin-beta-blocker-statin (BABS) score was calculated as previously described (Tapper et al. 2018). Handgrip strength was measured using a grip dynamometer and height-adjusted skeletal muscle mass index was assessed using a CT image at the level of the third lumbar vertebra (L3) (van der Werf et al. 2018; Derstine et al. 2018). Given the factors associated with OHE in geriatric cirrhosis, the sHE score was calculated based on hypoalbuminemia (≤ 3.5 g/dL) and hyperammonemia (≥ 80 µg/dL) as one point each, and patients were divided into three groups as follows: low (sHE = 0), intermediate (sHE = 1), and high (sHE = 2) risk (Miwa et al. 2022).

### Statistical analysis

Data are expressed as median and interquartile range for continuous variables and as numbers with percentages for categorical variables. Baseline characteristics were compared using the Mann-Whitney *U* test or chi-square test. With mortality as a competing risk, factors associated with OHE development were examined using Fine-Gray proportional hazards regression analysis. Results are presented as sub-distribution hazard ratios (SHRs) with 95% confidence intervals (CIs). The cumulative incidence of OHE was examined using Aalen–Johansen estimator. Cumulative incidence curves of OHE were created using the cumulative incidence function, and the groups were compared using Gray’s test. Multivariable analysis was performed, taking into account the multicollinearity of each variable. To compare the ability of sHE score with other variables, sensitivity and specificity was calculated.

A two-tailed *p* < 0.05 was used as the threshold for statistical significance. All statistical analyses were performed using JMP version 17.0.0 software (SAS Institute, North Carolina, USA) and R version 4.4.1 software (The R Foundation for Statistical Computing, Vienna, Austria).

## Results

### Clinical characteristics of enrolled patients with cirrhosis

Of the 378 patients screened, 270 met the eligibility criteria and were included in the analysis (Supplementary Fig. 2). The baseline characteristics of the patients are shown in Table 1. The included patients had a median age of 83 years, 63% were male, and 19% had ascites. The median Child-Pugh and MELD scores were 6 and 8 points, respectively. The sHE score 0:1:2 ratio was 107:129:34.

**Table 1:** Baseline characteristics of geriatric cirrhosis according to OHE development

Characteristic	All patients	OHE	No OHE	*p*-value*
	(*n* = 270)	(*n* = 41)	(*n* = 229)	
Age (years)	83 (81–86)	82 (81–85)	83 (81–86)	0.303
Male, n (%)	169 (63)	14 (34)	155 (68)	< 0.001
Body mass index (kg/m^2^)	22.5 (20.6–25.1)	23.9 (21.5–25.7)	22.3 (20.5–24.8)	0.150
Etiology (HCV/HBV/ALD/MASH/Others), n	118/13/25/8/106	18/1/1/2/19	10/12/24/6/87	0.378
Diabetes mellitus, n (%)	83 (31)	16 (39)	67 (29)	0.287
Ascites, n (%)	52 (19)	16 (39)	36 (16)	0.001
Hepatocellular carcinoma, n (%)	230 (85)	35 (85)	195 (85)	1.000
Child–Pugh score	6 (5–7)	7 (6–8)	5 (5–6)	< 0.001
MELD score	8 (7–9)	9 (7–10)	8 (7–9)	0.005
International normalized ratio	1.04 (0.97–1.11)	1.08 (1.01–1.17)	1.03 (0.96–1.10)	0.001
Platelet (10^9^/L)	115 (78–160)	106 (85–139)	116 (78–164)	0.456
Creatinine (mg/dL)	0.83 (0.60–1.10)	0.74 (0.66–0.87)	0.86 (0.70–1.05)	0.018
Albumin (g/dL)	3.5 (3.1–3.8)	3.1 (2.9–3.5)	3.5 (3.2–3.8)	< 0.001
Bilirubin (mg/dL)	0.8 (0.6–1.1)	1.1 (0.8–1.5)	0.7 (0.6–1.0)	< 0.001
Sodium (meq/L)	139 (137–141)	139 (136–140)	139 (138–141)	0.101
Ammonia (mcg/dL)	50 (37–66)	70 (55–100)	47 (36–62)	< 0.001
Skeletal muscle mass index (cm^2^/m^2^)	40.3 (36.0–44.7)	41.4 (37.3–45.8)	39.5 (36.0–44.4)	0.126
Handgrip strength (kg)	17.4 (8.7–23.5)	13.9 (0.0–19.6)	17.9 (10.9–24.2)	0.010
BCAA, n (%)	71 (26)	16 (39)	55 (24)	0.069
Lactulose, n (%)	20 (7)	6 (15)	14 (6)	0.111
Rifaximin, n (%)	3 (1)	2 (5)	1 (0)	0.091
Nonselective beta–blocker, n (%)	25 (9)	3 (7)	22 (10)	0.862
Statin, n (%)	18 (7)	2 (5)	16 (7)	0.874
sHE score 0/1/2, n (%)	107/129/34 (40/48/13)	6/21/14 (15/51/34)	101/108/20 (44/47/9)	< 0.001

Values are presented as numbers (percentages) or medians (interquartile range)

*Groups were compared using the chi-square test or Mann-Whitney *U* test

*ALD*, alcohol-related liver disease; *BCAA*, branched-chain amino acid; *HBV*, hepatitis B virus; *HCV*, hepatitis C virus; *MELD*, model for end-stage liver disease; *MASH*, metabolic dysfunction-associated steatohepatitis; *OHE*, overt hepatic encephalopathy; *sHE*, simple hepatic encephalopathy

### Comparison between patients with and without OHE development

A comparison between patients with and without OHE development is also shown in Table 1. Patients that developed OHE had a higher prevalence of female sex and ascites than those without. In addition, patients with OHE had significantly worse liver functional reserves based on the Child-Pugh score, MELD score, international normalized ratio, and albumin, bilirubin, and ammonia levels. In terms of sarcopenia, handgrip strength was significantly lower in patients with OHE than in those without. Consequently, patients who developed OHE had a worse sHE score than those who has not (sHE 0:1:2 of 6:21:14 vs. 101:108:20; *p* < 0.001).

### Independent factors for OHE development in geriatric cirrhosis

**Fig. 1:**
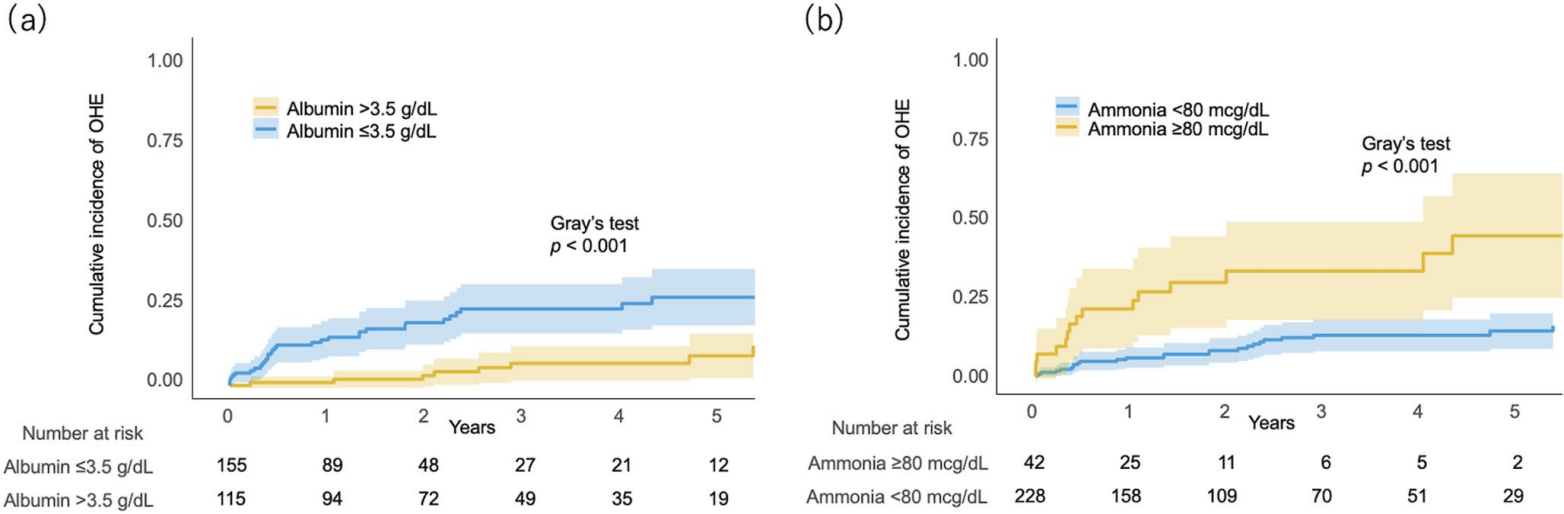
Cumulative incidence of OHE divided by serum **a** albumin and **b** ammonia levels

During a median follow-up of 1.8 years, 41 (15%) patients developed OHE and 120 (44%) patients died. Using the Aalen–Johansen estimator, the cumulative incidence of OHE during the observational period was 23%. The cumulative incidence of OHE at 1, 3, and 5 years was 9%, 16%, and 19%, respectively. Multivariable analysis was used to assess the factors associated with OHE development in geriatric cirrhosis (Table 2). After adjustment, male sex (SHR, 0.29; 95% CI, 0.13–0.63; *p* = 0.002), serum albumin (SHR, 0.51; 95% CI, 0.27–0.98; *p* = 0.042), and ammonia (SHR, 1.01; 95% CI, 1.00–1.02; *p* = 0.006) levels were independent factors associated with OHE development. Patients with hypoalbuminemia (SHR, 3.80; 95% CI, 1.80–8.02; *p* < 0.001) and hyperammonemia (SHR, 4.00; 95% CI, 2.15–7.43; *p* < 0.001) had a significantly higher cumulative incidence of OHE (Fig. 1a and b). Details of the univariable analysis are shown in Supplementary Table 1. The incidence of OHE according to lactulose and rifaximin therapy is detailed in Supplementary Table 2.

**Table 2:** Factors associated with OHE development in patients with geriatric cirrhosis

Characteristic	SHR (95% CI)	*p*-value*
Age (years)	0.97 (0.87–1.09)	0.638
Male	0.29 (0.13–0.63)	0.002
MELD score	1.07 (0.94–1.21)	0.297
Albumin (mg/dL)	0.51 (0.27–0.98)	0.042
Ammonia (mcg/dL)	1.01 (1.00–1.02)	0.006
Skeletal muscle mass index (cm^2^/m^2^)	1.00 (0.98–1.04)	0.574
Handgrip strength (kg)	0.99 (0.96–1.03)	0.773
Ascites	2.20 (1.01–4.76)	0.047

* Multivariable analysis was performed using the Fine–Gray competing risk regression model

*CI*, confidence interval; *MELD*, model for end-stage liver disease; *OHE*, overt hepatic encephalopathy; *SHR*, sub-distribution hazard ratio

### Impact of the sHE score on OHE development in geriatric cirrhosis

**Fig. 2:**
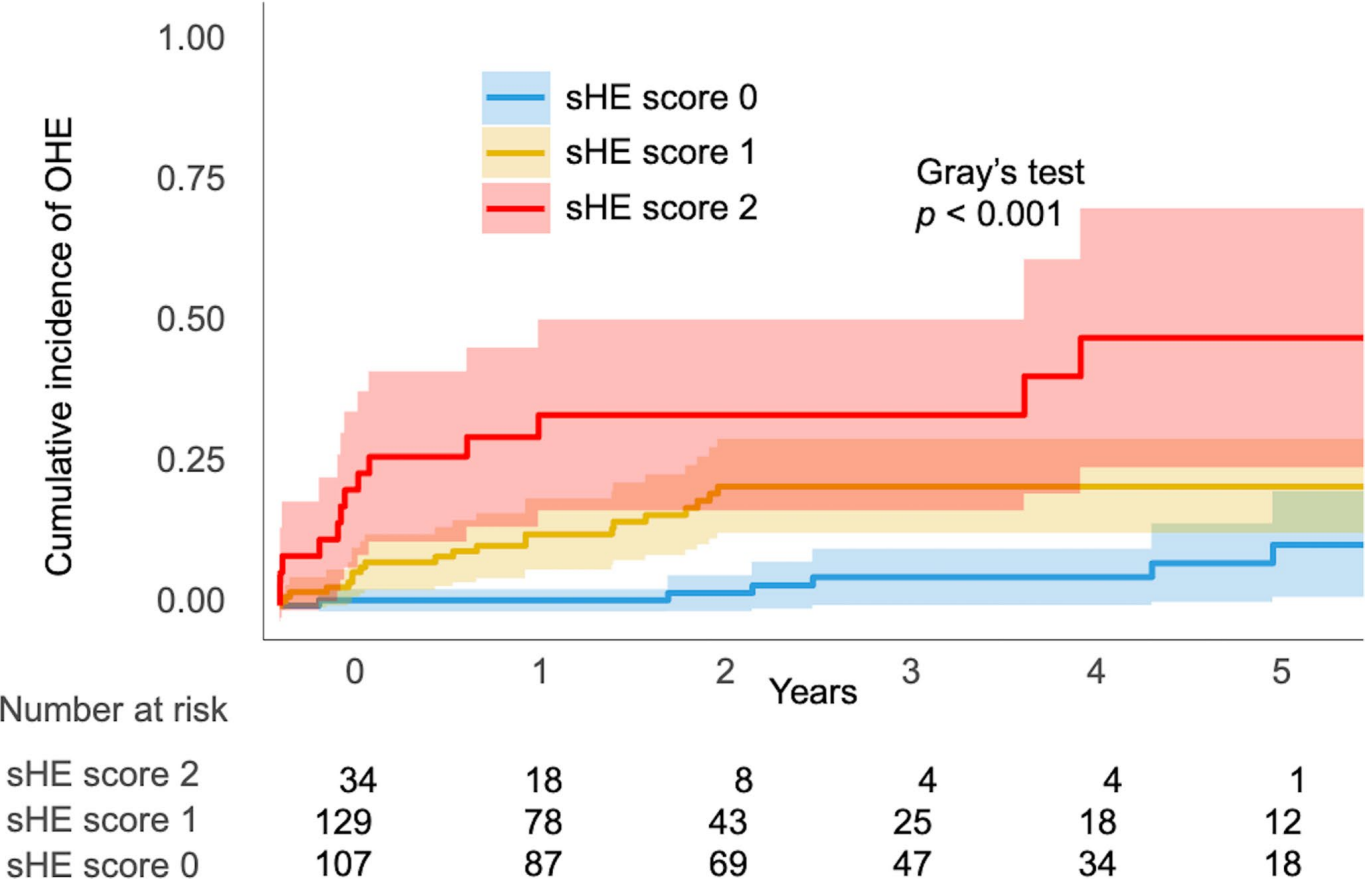
Cumulative incidence of OHE divided by serum **a** albumin and **b** ammonia levels OHE, overt hepatic encephalopathy

As serum albumin and ammonia levels were independent factors associated with OHE development in geriatric cirrhosis (Table 2), the impact of the sHE scores derived from these variables was also examined using multivariable analysis (Table 3). After adjustment, the sHE score (SHR, 2.18; 95% CI, 1.34–3.57; *p* = 0.002) was determined as an independent factor for OHE development in geriatric cirrhosis. The cumulative incidence of OHE at 1, 3, and 5 years was 1%, 5%, and 8% in the low-risk group; 10%, 21%, and 21% in the intermediate-risk group; and 28%, 35%, and 48% in the high-risk group, respectively (Fig. 2: *p* < 0.001). The risk of OHE development was significantly higher in the sHE high-risk group (SHR, 9.98; 95% CI, 3.97–25.07; *p* < 0.001), followed by the intermediate-risk group (SHR, 3.48; 95% CI, 1.44–8.45; *p* = 0.006), compared to those in the low-risk group.

**Table 3:** Impact of sHE score on OHE development in geriatric cirrhosis

Characteristic	SHR (95% CI)	*p*-value*
Age (years)	0.95 (0.85–1.07)	0.395
Male	0.33 (0.16–0.72)	0.005
MELD score	1.05 (0.93–1.19)	0.442
sHE score	2.18 (1.34–3.57)	0.002
Skeletal muscle mass index (cm^2^/m^2^)	1.01 (0.98–1.04)	0.616
Handgrip strength (kg)	0.99 (0.95–1.03)	0.633
Ascites	2.59 (1.29–5.21)	0.008

* Multivariable analysis was performed using the Fine–Gray competing risk regression model

*CI*, confidence interval; *MELD*, model for end-stage liver disease; *OHE*, overt hepatic encephalopathy; s*HE*, simple hepatic encephalopathy; *SHR*, sub-distribution hazard ratio

### Comparison between the ability of sHE score and other variables on predicting OHE development

The ability of each variable to identify the occurrence of OHE within 5-years is shown in Supplementary Table 3. An sHE score of ≥ 1 demonstrated a sensitivity of 87.2%, while an sHE score of 2 exhibited a high specificity of 90.9%. The ability of ascites and predetermined cutoff values of the BABS score to identify OHE within 5-years are also demonstrated.

## Discussion

With the increasing aging population in society, patients with cirrhosis are also experiencing associated increase in age (Enomoto et al. 2020, 2024; Louissaint et al. 2022). The development of OHE is a significant complication of cirrhosis and the prognosis of patients with OHE is poor (Rose et al. 2020). Considering the challenges in identifying CHE in geriatric cirrhosis with physiologically impaired neuropsychiatric function, it is crucial to establish a useful and rational tool to assess the risk of OHE development in this patient group. The primary finding of the present study was that serum albumin and ammonia levels were robust factors for the development of OHE in geriatric cirrhosis. The second finding was that the sHE score, comprising only serum albumin and ammonia levels, was a useful index for predicting the development of OHE in geriatric cirrhosis.

In the present study, patients with hypoalbuminemia had a 4-fold higher incidence of OHE than those with normal albumin levels. The pathophysiological role of albumin in HE can be explained by its association with inflammation. Inflammation correlates with the development and severity of HE (Claeys et al. 2021) and pro-inflammatory cytokines contribute to the development of cerebral edema, which is central to the pathogenesis of HE (Patidar and Bajaj 2015). Albumin infusion reduces inflammatory cytokines, endotoxins, and oxidative stress, and consequently improves HE in patients with cirrhosis (Wong and Loo 2023). In addition, increasing serum albumin levels by branched-chain amino acid supplementation is a promising treatment option for patients with cirrhosis associated HE (Gluud et al. 2017). The above reports support the results of the present study, indicating that serum albumin levels are a robust biomarker for the future development of OHE in geriatric cirrhosis. Indeed, serum albumin levels have been reported to stratify the risk of developing OHE in patients with cirrhosis in the United States and in Asia, which is consistent with our findings in the present study (Tapper et al. 2018; Bai et al. 2019; Miwa et al. 2022). Older patients with cirrhosis can easily develop malnutrition, which leads to protein deficiency and sarcopenia (Partridge et al. 2018). Therefore, it is essential to monitor serum albumin levels to stratify the risk of OHE and maintain it to improve the outcomes of older patients with cirrhosis.

In addition to hypoalbuminemia, hyperammonemia also plays an important role in the pathogenesis of HE. In the present study, patients with hyperammonemia had a 4-fold higher incidence of OHE than those within the normal range. Hyperammonemia contributes significantly to the development of HE through mitochondrial dysfunction, increased oxidative stress, and astrocyte swelling (Patidar and Bajaj 2015). Elevated serum ammonia levels were identified as a risk factor for the development of OHE in a multicenter cohort study (Ballester et al. 2023). In addition, serum ammonia levels are useful in predicting not only OHE but also hospitalization owing to other liver-related complications, including decompensated events (Tranah et al. 2022), and have a strong impact on mortality in patients with cirrhosis (Tranah et al. 2022; Miwa et al. 2024). In contrast, ammonia-lowering therapy is an essential treatment for OHE in these patients (Ridola et al. 2020). In patients with cirrhosis, hyperammonemia is caused by intestinal production, impaired hepatic urea cycle, decreased renal excretion, inadequate muscle detoxification, and portosystemic shunt (Gallego-Durán et al. 2024). These previous studies support the results of the present study, indicating that serum ammonia levels stratify the risk of OHE in geriatric cirrhosis.

Another important finding of the present study was the identification of a simple scoring model for the risk stratification of OHE in geriatric cirrhosis. The goal in this scoring system was to create a risk stratification model based solely on blood tests, using easily memorable cutoff values. In the present study, serum albumin and ammonia levels were independent factors for OHE development in geriatric cirrhosis, and the sHE score, composed of these factors, was also shown to be useful for predicting OHE development in the geriatric population. Several studies have reported that inclusion of serum albumin levels in scoring models is effective in predicting OHE development in patients with cirrhosis (Tapper et al. 2018; Labenz et al. 2019; Gil-Gómez et al. 2021). Furthermore, the combination of serum albumin and ammonia levels with other clinical variables can improve the prediction model for the development of OHE (Ballester et al. 2023). Although these models have a favorable ability to identify OHE development, they use complex scoring formulas for assessment and have limited clinical use in daily practice. Compared with these scoring systems, the advantages of the sHE score lie in its accessibility and memorability, allowing immediate decision making in daily practice. In particular, the incidence of OHE was notably low in patients with an sHE score of 0. As the risk of OHE development increases in geriatric cirrhosis patients with an sHE score ≥ 1, patients with either hypoalbuminemia or hyperammonemia require careful follow-up for HE. Of note, the MELD score did not predict OHE occurrence in our study and the ability of sHE score consisting only of biochemical parameters were better than ascites which requires medical imaging. Although the BABS score is a reliable indicator of OHE development, there was a marked imbalance between sensitivity and specificity with the predetermined cutoff value may be effective to stratify those with worse liver functional reserves (Tapper et al. 2018). Given its simplicity, the sHE score is an effective tool to stratify the risk of OHE in geriatric cirrhosis.

The present study acknowledges a few limitations. First, because this study focused on patients with cirrhosis in a specific region, the results may not be generalizable to other regions. Second, because this was a retrospective study, the possibility of bias cannot be entirely excluded. Third, female sex was identified as an independent risk factor for the development of OHE in geriatric cirrhosis, which is inconsistent with findings from a large cohort study (Tapper et al. 2018). The inclusion of only geriatric patients may have introduced a selection bias that affected the results of our study. Lastly, unmeasured variables and the treatment of HE may have potentially influenced the development of OHE in our study. Therefore, further prospective studies are necessary to verify the results of this study. However, we believe that the impact of these limitations is minimal because this study was conducted by reviewing a prospectively collected dataset comprising a sufficient number of patients, enabling a robust multivariable analysis.

In conclusion, the present study demonstrated that serum albumin and ammonia levels are associated with the development of OHE in geriatric cirrhosis. The sHE score is a useful index for predicting OHE development in older patients with cirrhosis. Further studies are required to provide a definitive solution to improve the impact of HE on geriatric cirrhosis..

## Supplementary Information

Below is the link to the electronic supplementary material.

ESM 1 (DOCX 126 KB)

ESM 2 (DOCX 28.8 KB)

ESM 3 (DOCX 19.9 KB)

## Data Availability

No datasets were generated or analysed during the current study.
