# Supplementary material for: Simple scoring model for predicting overt hepatic encephalopathy in geriatric cirrhosis: A multicenter retrospective cohort study
Source: Metab Brain Dis. 2025 Sep 10;40(7):263. doi: 10.1007/s11011-025-01691-x (PMC12423195; doi:10.1007/s11011-025-01691-x)
Supplement: Supplementary file 1 — (DOCX 126 KB) [file 11011_2025_1691_MOESM1_ESM.docx]

Supplementary Table 1. Univariable analysis of factors associated with OHE development in geriatric cirrhosis

| Characteristic | SHR (95% CI) | *p-*value* |
| --- | --- | --- |
| Age (years) | 0.97 (0.89–1.06) | 0.515 |
| Male | 0.29 (0.15–0.55) | <0.001 |
| Body mass index (kg/m^2^) | 1.04 (0.98–1.11) | 0.208 |
| Etiology |  |  |
| HCV^†^ | 1.00 |  |
| HBV | 0.58 (0.08–4.40) | 0.600 |
| ALD | 0.25 (0.03–1.88) | 0.180 |
| MASH | 2.24 (0.61–9.26) | 0.210 |
| Others | 1.44 (0.76–2.75) | 0.270 |
| Diabetes mellitus | 1.53 (0.82–2.85) | 0.185 |
| Ascites | 3.60 (1.92–6.73) | <0.001 |
| Hepatocellular carcinoma | 0.93 (0.41–2.11) | 0.858 |
| Child–Pugh score | 1.64 (1.33–2.03) | <0.001 |
| MELD score | 1.15 (1.05–1.26) | 0.004 |
| International normalized ratio | 16.40 (3.68–73.02) | <0.001 |
| Platelet (10^9^/L) | 1.00 (0.99–1.00) | 0.416 |
| Creatinine (mg/dL) | 0.35 (0.09–1.41) | 0.138 |
| Albumin (g/dL) | 0.39 (0.24–0.63) | <0.001 |
| Bilirubin (mg/dL) | 2.63 (1.92–3.61) | <0.001 |
| Sodium (meq/L) | 0.91 (0.83–1.00) | 0.041 |
| Ammonia (mcg/dL) | 1.01 (1.01–1.02) | <0.001 |
| Skeletal muscle mass index (cm^2^/m^2^) | 1.01 (0.99–1.04) | 0.261 |
| Handgrip strength (kg) | 0.97 (0.94–0.99) | 0.018 |
| BCAA | 2.10 (1.12–3.948) | 0.021 |
| Lactulose | 2.54 (1.04–6.21) | 0.041 |
| Rifaximin | 9.91 (1.10–89.05) | 0.041 |
| Nonselective beta–blocker | 0.83 (0.26–2.66) | 0.749 |
| Statin | 0.85 (0.24–2.97) | 0.800 |
| sHE score |  |  |
| 0^†^ | 1.00 |  |
| 1 | 3.48 (1.44–8.45) | 0.006 |
| 2 | 9.98 (3.97–25.07) | <0.001 |

* Univariable analyses were performed using the Fine-Gray competing risk regression model.

†Reference group.

Abbreviations: ALD, alcohol-related liver disease; BCAA, branched-chain amino acid; CI, confidence interval; HBV, hepatitis B virus; HCV, hepatitis C virus; MELD, model for end-stage liver disease; MASH, metabolic dysfunction-associated steatohepatitis; OHE, overt hepatic encephalopathy; sHE, simple hepatic encephalopathy; SHR, sub-distribution hazard ratio; HR, hazard ratio.
