# Supplementary material for: Simple scoring model for predicting overt hepatic encephalopathy in geriatric cirrhosis: A multicenter retrospective cohort study
Source: Metab Brain Dis. 2025 Sep 10;40(7):263. doi: 10.1007/s11011-025-01691-x (PMC12423195; doi:10.1007/s11011-025-01691-x)
Supplement: Supplementary file 2 — (DOCX 28.8 KB) [file 11011_2025_1691_MOESM2_ESM.docx]

Supplementary Table 2. OHE development according to lactulose and rifaximin therapy

| Characteristic | OHE (%) |
| --- | --- |
| Lactulose | 30.0 |
| No lactulose | 14.0 |
| Rifaximin | 66.7 |
| No Rifaximin | 14.6 |

Abbreviations: OHE, overt hepatic encephalopathy.
