# Supplementary material for: Simple scoring model for predicting overt hepatic encephalopathy in geriatric cirrhosis: A multicenter retrospective cohort study
Source: Metab Brain Dis. 2025 Sep 10;40(7):263. doi: 10.1007/s11011-025-01691-x (PMC12423195; doi:10.1007/s11011-025-01691-x)
Supplement: Supplementary file 3 — (DOCX 19.9 KB) [file 11011_2025_1691_MOESM3_ESM.docx]

Supplementary Table 3. Sensitivity and specificity of each variable for the development of OHE within 5-years

| Characteristic | Sensitivity (%) | Specificity (%) |
| --- | --- | --- |
| Ascites | 41.0 | 84.4 |
| MELD score ≥ 15 | 0.0 | 96.5 |
| sHE score ≥ 1 | 87.2 | 44.2 |
| sHE score 2 | 33.3 | 90.9 |
| BABS score ≥ -9 | 100.0 | 0.0 |
| BABS score ≥ 21 | 0.0 | 100.0 |

Abbreviations: BABS, bilirubin–albumin–beta–blocker–statin; MELD, Model for End-Stage Liver Disease; sHE, simple hepatic encephalopathy.
